# CD24 ^+^ MDSC-DCs Induced by CCL5-Deficiency Showed Improved Antitumor Activity as Tumor Vaccines

**DOI:** 10.1055/s-0042-1743569

**Published:** 2022-03-08

**Authors:** Lei Huang, Zequn Ding, Yan Zhang

**Affiliations:** 1 State Key Laboratory of Oncogenes and Related Genes, Renji-Med-X Stem Cell Research Center, Ren Ji Hospital, School of Biomedical Engineering, Shanghai Jiao Tong University, Shanghai, People's Republic of China; 2 Med-X Research Institute, School of Biomedical Engineering, Shanghai Jiao Tong University, Shanghai, People's Republic of China

**Keywords:** CD24+ MDSC-DCs, CCL5-deficiency, tumor vaccines, antitumor activity, Tregs

## Abstract

**Background** Dendritic cell (DC) tumor vaccine has been extensively utilized in preclinical and clinical studies; however, this technique has encountered many difficulties, particularly in late-stage tumor patients. For those, ex vivo-induced DCs are actuallymyeloid-derived suppressive cells-derived DCs (MDSC-DCs). MDSCs with immunosuppressive activity, but not monocytes, became the major DC precursor. Thus, how to enhance antitumor activity of MDSC-DCs is urgent need to address.

**Methods** We utilized 4T1 and MC38 tumor-bearing both wildtype and CC chemokine ligand 5 ^−/−^ (CCL5 ^−/−^ ) mice as animal models. MDSC-DCs were induced from splenocytes of these mice by granulocyte macrophage–colony stimulating factor/interleukin-4 with or without all-trans-retinoic acid (ATRA) in vitro for 7 days, then incubated with tumor-cell-lysis to treat mouse models for total three doses. For human MDSC-DCs, peripheral bloods from colorectal cancer patients were induced in vitro as murine cells with or without T- lymphocytes depletion to get rid of CCL5.

**Results** Flow cytometry analysis showed that MDSCs from *CCL5* ^−/−^ mice could be induced into a new type of CD24 ^+^ MDSC-DCs in the presence of ATRA, which had more antitumor activity than control. Antibody blocking and adoptive transfer experiments demonstrated that downregulation of regulatory T cells (Tregs) mediated the inhibition of CD24 ^+^ MDSC-DCs on tumor growth. Mechanically, CD24 ^+^ MDSC-DCs inhibited Tregs' polarization by secreting cytokine or coactivators' expression. What's important, decreasing CCL5 protein levels by T- lymphocytes depletion during both murine and human MDSC-DCs in vitro induction could also acquire CD24 ^+^ MDSC-DCs.

**Conclusion** Knockdown of CCL5 protein during MDSC-DCs culture might provide a promising method to acquire DC-based tumor vaccines with high antitumor activity.

## Introduction

Cellular immunotherapy, which using immune cells to eliminate cancer, changes the outlook for cancer patients. Immune checkpoint inhibition, Chimeric antigen receptor T cells therapy, and dendritic cell (DC)-based cancer vaccine are now emerging immune-related treatments for many malignancies. 1 2 3 DCs are the most important antigen presenting cells and have been categorized as different subtypes that are conventional DC (cDC), plasmacytoid DC (pDC), and monocyte-derived DC (MoDC). 4 To take advantage of its features, personalized DC-based vaccines have been extensively utilized in numerous preclinical and clinical studies. 5 6 In 2010, Sipuleucel-T became the first approved DC cancer vaccine for the treatment of late-stage prostate cancer. 7 8 Although Sipuleucel-T has not been very widely used, it demonstrated how the safety and immunogenicity DC-based vaccine would be. 9 10

The DCs for tumor vaccines are usually manufactured by inducing monocytes or hematopoietic stem and progenitor cells from cancer patients' peripheral blood by recombinant granulocyte macrophage–colony stimulating factor (GM-CSF) and interleukin-4 (IL-4) cytokines and subsequently treated with tumor-associated antigens to acquire antitumor activity. 11 12 13 However, along with tumor progression, inflammatory cytokines and chemokines exert tumor-promoting effect by “educating” normal immune cells to become immunosuppressive types. 14 15 16 As our and other groups' data showed that a lot of myeloid-derived suppressive cells (MDSCs) that are heterogeneous immature myeloid cells with immunosuppressive activity, but not monocytes, accumulated in cancer patients' peripheral blood, especially in patients with advanced cancer. 17 18 19 Therefore, DCs ex vivo differentiation from monocytes of peripheral blood in tumor patients are actually MDSC-derived DCs (MDSC-DCs). Thus, how to improve the antitumor activity of ex vivo differentiated DCs is undoubtedly the focus of DC-based tumor vaccine research.

Our previous studies showed that CC chemokine ligand 5 (CCL5), which is associated with poor prognosis and metastasis in many types of tumors, 20 plays an important role in myeloid cell's differentiation. 21 22 So, we wondered if CCL5 involves in maintaining the immunosuppressive capacity of MDSCs and if CCL5-deficiency in MDSCs could make MDSC-DCs into a different phenotype. In this project, we figured out the antitumor activity of MDSC-DCs as tumor vaccines and tried to interrupt *CCL5* gene in MDSC-DCs to enhance their antitumor activity. CCL5-deficiency induced the formation of CD24 ^+^ MDSC-DCs and these cells showed enhanced antitumor abilities via inhibiting differentiation of CD4 ^+^ T cells into regulatory T cells (Tregs). Our research provided a new method for the development of DC-based cancer vaccines.

## Results

### DCs Derived from CCL5-deficiency MDSCs Showed High CD24 Expression

Plenty of research have proved that immune cells in tumor bearing mice's spleen can basically mirror the situation of cancer patients' peripheral blood. 17 23 24 To choose the suitable tumor-bearing murine models to mimic the higher proportion of MDSCs accumulated in peripheral blood at late stage of tumor patients, we first constructed eight different models of prostatic cancer (TRAMP-C1), breast cancer (4T1,DA3), colon cancer (CT26, MC38), melanoma (B16F10), and sarcoma (MethA,C3) developed in BALB/C or C57BL/6 mice and examined the proportion of CD11b ^+^ Gr-1 ^+^ MDSCs in spleens by FACS after 21 days of tumor cells inoculation. Compared with control mice, the percentages of MDSCs in spleens were significantly increased in all tumor-bearing murine models, particularly in 4T1-Balb/c and MC38-C57B6 models ( Supplementary Fig. S1A and S1B [available in the online version only]). Thus, in our following experiments we utilized splenocytes from these two tumor-bearing murine models as initial cells to induce MDSC-DCs with GM-CSF and IL-4.

**Fig. 1:**
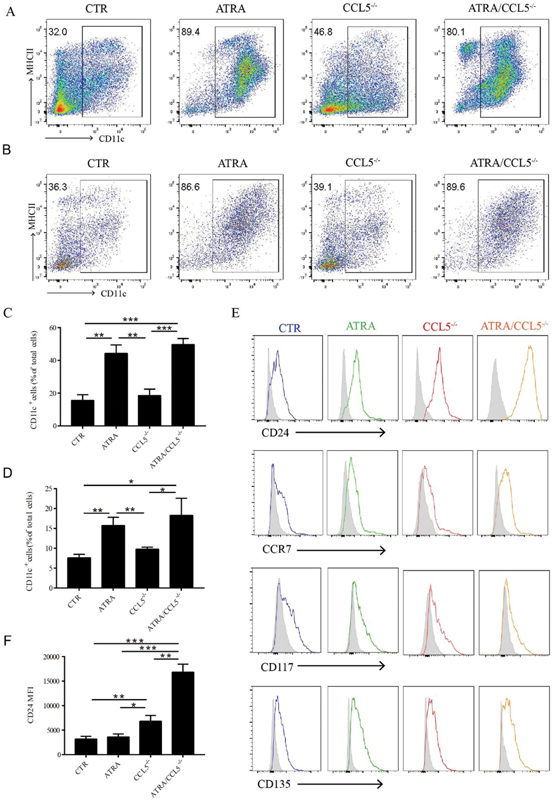
Knockout of CC chemokine ligand 5 (CCL5) induced the formation of CD24 ^+^ myeloid-derived suppressive cells-derived DCs (MDSC-DCs) derived from splenocytes of tumor-bearing mice. ( **A** ) Representative FACS plots of differentiated MDSC-DCs derived from 4T1 tumor mice model. ( **B** ) Representative FACS plots of differentiated MDSC-DCs derived from MC38 tumor mice model. ( **C** ) The percentage of CD11c ^+^ MDSC-DCs derived from splenocytes of 4T1 tumor-bearing mice in different culture systems. ( **D** ) The percentage of CD11c ^+^ MDSC-DCs derived from splenocytes of MC38 tumor-bearing mice in different culture systems. ( **E** ) Representative expression of the indicated surface markers on gated CD11c ^+^ MDSC-DCs from 4T1 tumor-bearing mice splenocytes cultures. The isotype monoclonal antibody of indicated markers was included as controls. ( **F** ). CD24 expression in MDSC-DCs derived from different culture methods. The experiments were repeated *n* = 3 for each group. * *p* < 0.05, ** *p* < 0.01, *** *p* < 0.001. Data were represented as mean ± standard deviation.

Our results showed that the differentiation efficiency of MDSCs into CD11c ^+^ MDSC-DCs in the presence of GM-CSF and IL-4 was low, around 30%, in both 4T1-Balb/c and MC38-C57B6 models ( Fig. 1A–D ), which were consistent with the data reported by other groups. 5 25 Since all-trans-retinoic acid (ATRA) has been shown to have a strong effect on myeloid cell differentiation, 26 27 we added 1.5uM ATRA into the MDSC-DCs culture medium with GM-CSF plus IL-4 and, as expected, the production of MDSC-DCs significantly increased to around 80% in the new culture system, independent of CCL5 protein ( Fig. 1A–D ). For CCL5-deficiency MDSC-DCs, we constructed tumor models developed in CCL5-knockout mice and induced MDSCs via GM-CSF and IL-4 into CCL5 ^−/−^ MDSC-DCs. To further characterize MDSC-DCs obtained from different origins and culture systems, we examined expression levels of several DC markers, such as CD24, CCR7, CD117, and CD135 on the MDSC-DCs in 4T1-tumor bearing mice. FACS results showed that the expression of CCR7, CD117, and CD115 among these MDSC-DCs did not have notable changes, whereas CCL5-deficiency MDSC-DCs expressed higher amount of CD24 compared with the other three cohorts ( Fig. 1E and F ). MDSC-DCs in MC38 tumor-bearing mice also showed similar expression level of DC markers as MDSCs in 4T1 tumor-bearing mice ( Supplementary Fig. S2A and S2B , available in the online version only). Collectively, our results showed that ATRA could enhance the induction-efficiency of MDSCs into MDSC-DCs and DCs derived from CCL5-deficiency MDSCs showed higher CD24 expression.

**Fig. 2:**
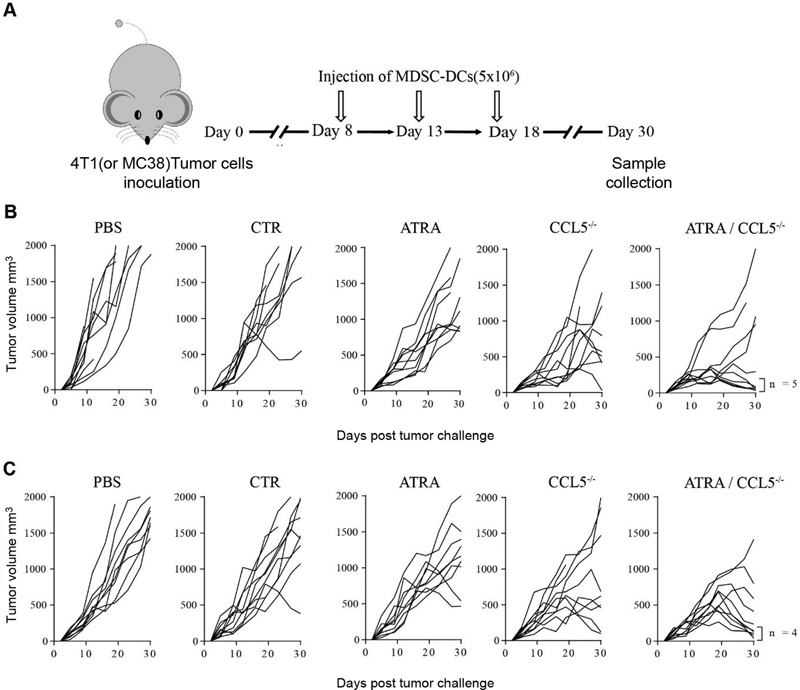
CC chemokine ligand 5 (CCL5)-deficiency myeloid-derived suppressive cells-derived DCs (MDSC-DCs)-based tumor vaccine had potent antitumor activity. ( **A** ). Flowchart of MDSC-DC vaccines preparation and treatment plan. ( **B** ) Tumor growth curves of 4T1 tumor-bearing mice treated with different groups of MDSC-DC vaccines. Tumor growth was monitored every 2 to 3 days. *n* = 10 mice per group. ( **C** ) Tumor growth curves of MC38 tumor-bearing mice treated with different groups of MDSC-DC vaccines. Tumor growth was monitored every 2 to 3 days. *n* = 10 mice per group. PBS, phosphate buffered saline.

### CCL5-Deficiency MDSC-DCs-Based Tumor Vaccines Had Potent Antitumor Activity

CD24 is a cell surface heavily glycosylated glycosylphosphatidylinositol-anchored protein not only expressed in many cancers but also showed in adaptive immune responses. 28 CD24 is a redundant costimulatory molecule expressed on variety of antigen-presenting cells, including DCs and has been recognized as general DC marker for some subsets of mouse DCs. 4 Previous studies have reported that CD24 expression determines antigen presenting ability of DCs 29 and CD24 expression on DCs may also influence the T cell differentiation and activation. 30 31

To determine whether MDSC-DCs derived from different conditions have differential antitumor abilities, we utilized MDSC-DCs as tumor vaccines in both MC-38 colorectal cancer (CRC) and 4T1 breast cancer models. We used the freeze-thaw processing to get whole tumor cell lysates as a source of all potential antigens for antigen-loading and lipopolysaccharide (LPS) for maturation. Mice at background of BALB/c or C57BL/6 were inoculated subcutaneously with 4T1 or MC38 cells, respectively, and, after 7 days for tumor establishment, 5 × 10 ^6^ MDSC-DCs removed cell debris and death cells ( Supplementary Fig. S3A and S3B [available in the online version only]) were injected intravenously into tumor-bearing mice every 5 days for three times, whereas control mice received phosphate buffered saline (PBS) only ( Fig. 2A ). The tumor growth curves showed that tumor lysate-pulsed MDSC-DCs in either regular or ATRA-plus culture medium only elicited subtle antitumor activity compared with PBS group ( Fig. 2B and C ). However, CCL5 ^−/−^ MDSC-DCs, particular in the present of ATRA, had dramatically tumor-inhibitory effects on both 4T1 and MC38 tumor-bearing models ( Fig. 2B and C ). Collectively, CCL5-deficiency could enhance the antitumor activity of MDSC-DCs as tumor vaccine and ATRA could not only increase the differentiation of MDSC-DCs but also have a synergistic effect with CCL5-deficiency on boosting the tumor inhibition activity of MDSC-DCs.

### The Composition of Tumor-Infiltrated Leukocytes (TILs) Was Changed in CD24 ^+^ MDSC-DCs Tumor Vaccine-Treated Mice

**Fig. 3:**
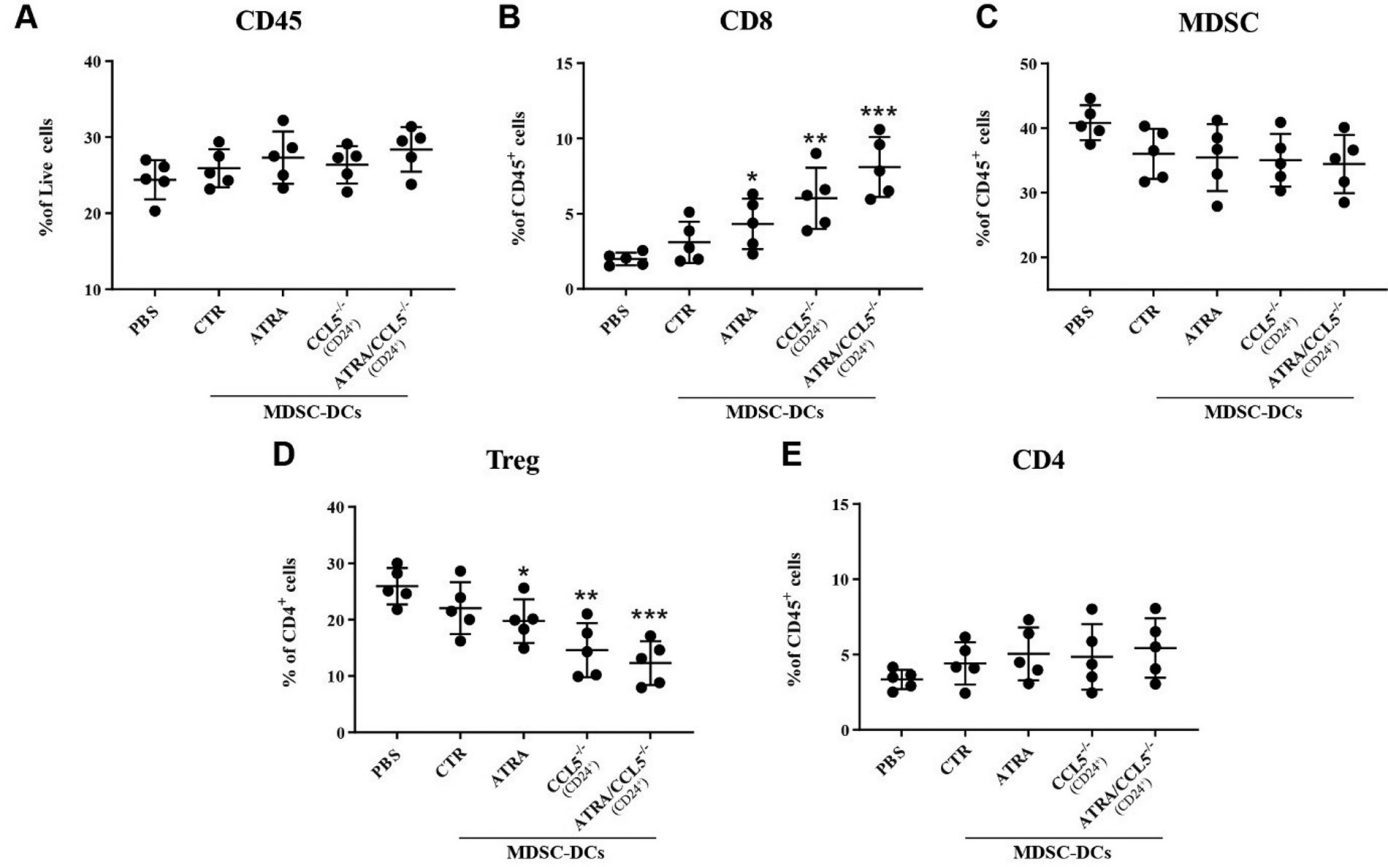
The tumor immune microenvironment was changed after CD24 ^+^ myeloid-derived suppressive cells-derived DCs (MDSC-DCs) vaccines treatment. ( **A** ) FACS qualification of tumor infiltrated CD45 ^+^ cells in different treatment groups. *n* = 5 mice per group. ( **B** ) FACS qualification of tumor infiltrated CD8 ^+^ cells in different treatment groups. *n* = 5 mice per group. ( **C** ) FACS qualification of tumor infiltrated Gr-1 ^+^ CD11b ^+^ cells in different treatment groups. *n* = 5 mice per group. ( **D** ) FACS qualification of tumor infiltrated CD4 ^+^ FOXP3 ^+^ cells in different treatment groups. *n* = 5 mice per group. ( **E** ) FACS qualification of tumor infiltrated CD4 ^+^ cells in different treatment groups. *n* = 5 mice per group. All the significance in the plot were compared with phosphate buffered saline (PBS) group. * *p* < 0.05, ** *p* < 0.01, *** *p* < 0.001. Data were represented as mean ± standard deviation.

In the following study, we defined CCL5 ^−/−^ MDSC-DCs with ATRA as CD24 ^+^ MDSC-DCs to distinguish CCL5-deficiency MDSC-DCs with or without ATRA. To further understand the mechanism involved in MDSC-DCs vaccine immunization, we analyzed by flow cytometry the major subsets of TILs, which are highly relevant to the regulation of tumor growth, 32 in 4T1 tumor-bearing mice treated with different MDSC-DCs vaccines. Fig. 3A and Supplementary Fig. S4A (available in the online version only) showed that the percentage of CD45 ^+^ TILs was slightly increased in mice vaccinated MDSC-DCs, compared with PBS group, but no significant difference among them. Considering the prominent role of CD8 ^+^ T cells in antitumor effect, 33 we first analyzed tumor-infiltrated CD3 ^+^ CD8 ^+^ CD4 ^-^ cells. All the MDSC-DCs vaccines treated mice had significantly increased frequency of tumor-infiltrating CD8 ^+^ T cells, particularly CD24 ^+^ MDSC-DCs, compared with PBS-treated control ( Fig. 3B and Supplementary Fig. S4B [available in the online version only]). Although CD8 ^+^ T cells can directly kill cancer cells, some other regulatory immune cells, such as MDSCs and Tregs, play important roles on regulation of antitumor activity of CD8 ^+^ T cells. 34 35 FACS results showed that the percentages of MDSCs in MDSC-DCs-treated-groups were only slightly lower than that of the PBS group, but there was no statistical difference ( Fig. 3C and Supplementary Fig. S4C [available in the online version only]), suggesting that inhibition of MDSCs might not contribute to the enhanced antitumor activity of CD24 ^+^ MDSC-DCs.

Tregs that also demonstrated strong ability in modulating CD8 ^+^ T cell function 36 and the results showed that the frequency of Tregs (CD4 ^+^ FOXP3 ^+^ ) cells in the PBS group were notably higher than MDSC-DCs-treated-groups, particularly the proportion of Tregs in CD24 ^+^ MDSC-DCs-treated-group ( Fig. 3D and Supplementary Fig. S4D [available in the online version only]), suggesting that low number of Tregs might mediate the enhanced antitumor activity of CD24 ^+^ MDSC-DCs. Since Tregs are subsets of CD4+ T cells, next we examined the population of CD3 ^+^ CD4 ^+^ T cells in TILs by FACS, and the results showed that there was no considerable divergence in CD4 ^+^ T cells between each group ( Fig. 3E and Supplementary Fig. S4E [available in the online version only]). These results suggested that polarization of Tregs might be changed in CD24 ^+^ MDSC-DCs-treated-group.

### Tregs Mediated the Antitumor Activity of CD24 ^+^ MDSC-DCs

**Fig. 4:**
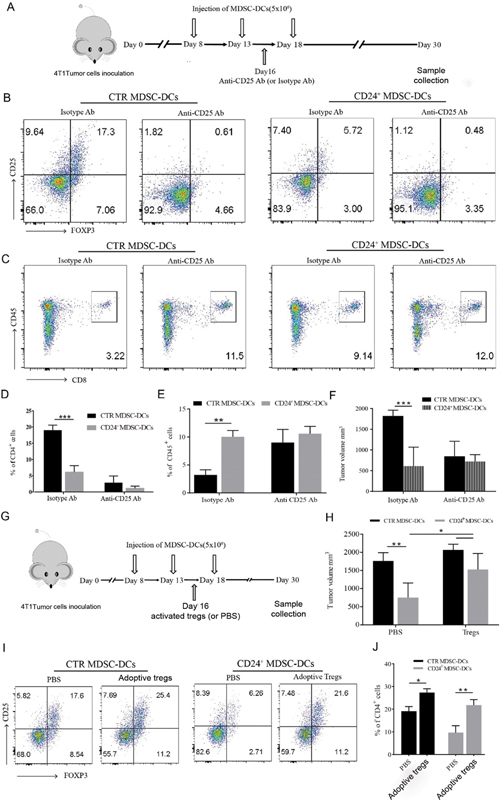
Regulatory T cells (Tregs) mediated the antitumor activity of CD24 ^+^ myeloid-derived suppressive cells-derived DCs (MDSC-DCs). ( **A** ) Flowchart of anti-CD25 monoclonal antibody (mAb) neutralization in MDSC-DCs vaccines treated mice. ( **B** ) Representative staining plots of tumor infiltrated Tregs after MDSC-DCs vaccines treatment with/without anti-CD25 mAb treated. ( **C** ) Representative staining plots of tumor infiltrated CD8 ^+^ T cells after MDSC-DCs vaccines treatment with/without anti-CD25 mAb treated. ( **D** ) FACS qualification of tumor infiltrated Tregs in different treatment groups. *n* = 5 mice per group. ( **E** ) FACS qualification of tumor infiltrated CD8 ^+^ T cells in different treatment groups. *n* = 5 mice per group. ( **F** ) Tumor volume of tumor-bearing mice after MDSC-DCs vaccines treatment with/without anti-CD25 mAb treated. *n* = 5 mice per group. ( **G** ) Flowchart of adoptive Tregs transfer in MDSC-DCs vaccines treated mice. ( **H** ) Tumor volume of tumor-bearing mice after MDSC-DCs vaccines treatment with/without adoptive Tregs transfer. *n* = 5 mice per group. ( **I** ). Representative staining plots of tumor infiltrated Tregs after MDSC-DC vaccines with/without adoptive Tregs transfer. ( **J** ) FACS qualification of tumor infiltrated Tregs after MDSC-DC vaccines with/without adoptive Tregs transfer. *n* = 5 mice per group. * *p* < 0.05, ** *p* < 0.01, *** *p* < 0.001. Data were represented as mean ± standard deviation. PBS, phosphate buffered saline.

To further investigate the Tregs' role in antitumor activity of CD24 ^+^ MDSC-DCs, 4T1 tumor-bearing mice were given three doses of CD24 ^+^ MDSC-DCs or control MDSC-DCs cultured with ATRA as tumor vaccines from 7 days after tumor inoculation with 5-day intervals. Anti-CD25 neutralizing antibody (PC61) that has been demonstrated to efficiently deplete Treg cells in vivo 37 or isotype antibody was intraperitoneally injected into mice 16 days after tumor inoculation and tumor samples were collected at 30 days for FACS analysis ( Fig. 4A ). FACS showed that there was a significant scavenging effect on tumor-infiltrated Tregs after anti-CD25 antibody injection in control (CTR) and CD24 ^+^ MDSC-DCs groups, although the number of Tregs was low in CD24 ^+^ MDSC-DCs-treated mice ( Fig. 4B and 4D ). In control (CTR) MDSC-DCs groups, the proportion of infiltrated CD8 ^+^ T cells increased significantly after anti-CD25 treatment compared with isotype antibody treatment, whereas in the CD24 ^+^ MDSC-DCs groups, the percentage difference between anti-CD25 monoclonal antibody (mAb) and isotype antibody was inconspicuous ( Fig. 4C and E ), which reflected the tendency on tumor growth of these mice. Fig. 4F showed that anti-CD25 neutralizing mAb (PC61) significantly inhibited tumor growth in control (CTR) MDSC-DCs groups, while there was no notable change on tumor growth between anti-CD25 neutralizing antibody and isotype antibody in CD24 ^+^ MDSC-DCs vaccinated mice, suggesting that intratumoral Tregs might contribute to the antitumor effect of CD24 ^+^ MDSC-DCs vaccines.

To further confirm these results, 1 × 10 ^6^ isolated Treg cells were transferred into the 4T1 tumor-bearing mice at 16 day after tumor challenge ( Fig. 4G ). The frequency of infiltrated Treg increased notably in both groups ( Fig. 4I ). Tumor volume increased in both groups after receiving Tregs, particularly in CD24 ^+^ MDSC-DCs groups and the difference on tumor volume between the two groups was reduced after receiving Treg reinfusion ( Fig. 4H ). Collectively, CD24 ^+^ MDSC-DCs achieved tumor growth inhibition by reducing the proportion of Tregs in tumor microenvironment (TME), which alleviated the immune suppression of the TME.

### CD24 ^+^ MDSC-DCs Inhibited Tregs' Polarization

**Fig. 5:**
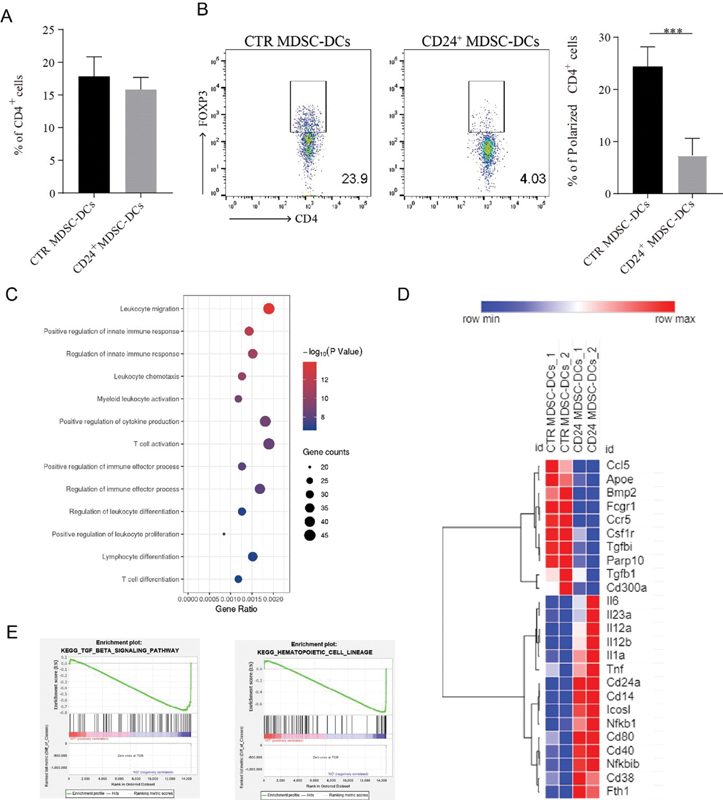
The genotype and phenotype of CD24 ^+^ myeloid-derived suppressive cells-derived DCs (MDSC-DCs). ( **A** ) FACS qualification of the proportion of regulatory T cells (Tregs) in the peripheral blood of MDSC-DCs vaccines treated 4T1 tumor-bearing mice. ( **B** ) MDSC-DCs coculture with naïve CD4 ^+^ T cells isolated from OT-II mice in the presence of 10 ng/mL Ovalbumin _323–339_ peptide and 5 ng/mL transforming growth factor-β1 for 4 days. The ratio of MDSC-DCs and naïve CD4 ^+^ T cells is 1 to 10. After 4 days cultured, the percentage of Tregs was analyzed by FACS. Representative staining plots of Tregs after 4-day coculture. ( **C** ) FACS qualification of the proportion of Tregs in the coculture system. The coculture experiment was repeated, *n* = 3 for each group. ( **D** ) Gene Ontology (GO) enrichment terms of differentially expressed regulation of immune response and T cell activation biological process derived from RNA-sequencing. ( **E** ) Expression of CD4 ^+^ T cell polarization-related and DCs function-related genes. ( **F** ) Gene Set Enrichment Analysis (GSEA) analysis of control (CTR) MDSC-DCs and CD24 ^+^ MDSC-DCs.

To figure out the mechanism on the reduced quantity of Tregs in CD24 ^+^ MDSC-DCs vaccine-treated mice, we first examined the number of Tregs in peripheral blood of control (CTR) and CD24 ^+^ MDSC-DCs groups in 4T1-bearing mice. And there was no significantly difference on the percentage of Tregs in peripheral blood between these two groups ( Fig. 5A ) suggesting that the migration of Tregs did not contribute to the lower level of Tregs in CD24 ^+^ MDSC-DCs vaccine mice. Next, we wondered if CD24 ^+^ MDSC-DCs had an effect on the polarization of Tregs in TME.

We set up an antigen presentation assay by coculturing control (CTR) or CD24 ^+^ MDSC-DCs with microbeads-isolated naïve OT-II CD4 ^+^ CD62L ^+^ T cells in the presence of Ovalbumin _323–339_ peptide and transforming growth factor-β1 (TGF-β1). After 4 days of coculture, control (CTR) MDSC-DCs induced significant higher percentage of Tregs than CD24 ^+^ MDSC-DCs ( Fig. 5B and C ), indicating that CD24 ^+^ MDSC-DCs inhibited T cells polarization to Tregs in TME. To further explore the molecular mechanism on the inhibitory effect of CD24 ^+^ MDSC-DCs on Tregs' polarization, the gene expressions of control (CTR) MDSC-DCs and CD24 ^+^ MDSC-DCs were examined by RNA-seq and Gene Ontology (GO) enrichment analysis showed that the main genes expression differences were distributed in innate immunity regulation, cytokine production, and T cell activation ( Fig. 5D ). Heatmap showed that CD24 ^+^ MDSC-DCs had a high expression of inhibiting Treg polarization relevant genes (Il12a, Il12b, Il6, Il23a, Il1a) and T cell activation relevant genes ( *Nfkb1, Tnf, Cd80, Cd40, Cd24a, CD14, Icosl, Fth1* ), while control (CTR) MDSC-DCs expressed higher level of genes related to Tregs differentiation and Tregs function ( *Apoe, Fcgr1, Tgfb1, Tgfbi, Parp10, Cd300a* ) ( Fig. 5E ). What's more, Gene Set Enrichment Analysis (GSEA) revealed that the percentage of Treg downregulation was negatively associated with TGF-β signaling activity in MDSC-DCs ( Fig. 5F ). Collectively, these results suggested that CD24 ^+^ MDSC-DCs might inhibit polarization of Tregs by secreting cytokines and expressing coactivators.

### Decreasing CCL5 Protein in Culture Medium during In Vitro Differentiation Could also Induce Similar Cells as CD24 ^+^ MDSC-DCs

**Fig. 6:**
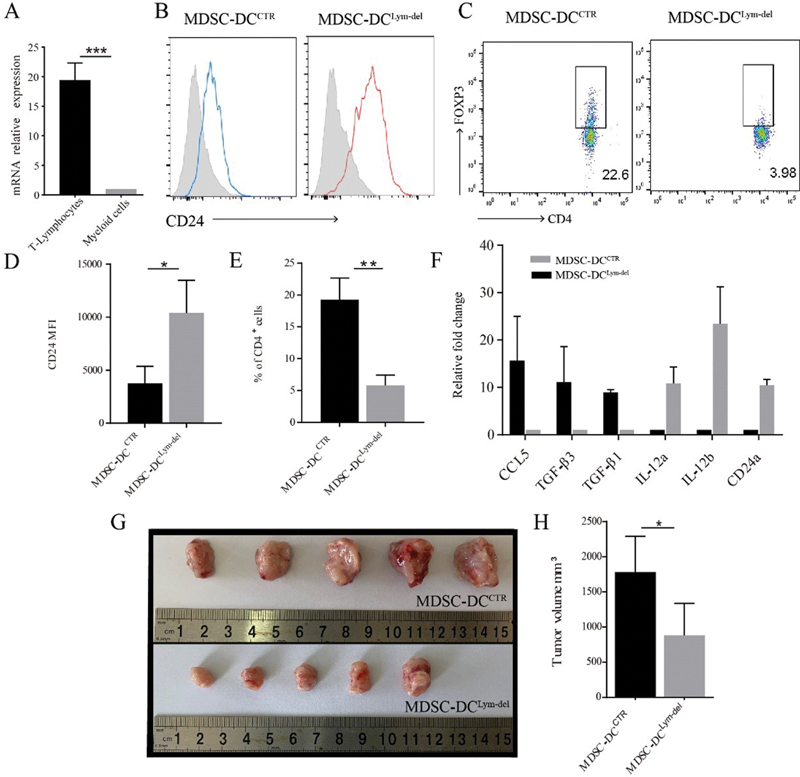
Lymphocytes depletion from tumor-bearing mice splenocytes induced CD24 ^+^ myeloid-derived suppressive cells-derived DCs (MDSC-DCs). ( **A** ) Relative gene expression of CC chemokine ligand 5 (CCL5) in T-lymphocytes and myeloid cells isolated from 4T1 tumor-bearing mice was examined by reverse-transcription polymerase chain reaction (RT-PCR). ( **B** ) Representative histogram plots for expression of CD24 on gated CD11c ^+^ MDSC-DCs. ( **C** ) Mean Fluorescence Intensity (MFI) of CD24 in MDSC-DCs derived from lymphocytes depletion and control group. ( **D** ) MDSC-DCs coculture with naïve CD4 ^+^ T cells isolated from OT-II mice in the presence of 10 ng/mL Ovalbumin _323–339_ peptide and 5 ng/mL transforming growth factor-β1 (TGF-β1) for 4 days. The ratio of MDSC-DCs and naïve CD4 ^+^ T cells is 1 to 10. After 4-day cultured, the percentage of Tregs was analyzed by FACS. Representative staining plots of Tregs after 4-day coculture. ( **E** ) FACS qualification of the proportion of Treg cells in the coculture system. ( **F** ) The expression of *CCL5* , *TGFβ1* , *TGFβ3* , *IL-12a* , *IL-12b,* and *CD24a* in control and MDSC-DCs ^Lym-del^ were analyzed by RT-PCR. ( **G** ) The tumor volume was measured after control and MDSC-DCs ^Lym-del^ vaccines treated. ( **H** ) Representative pictures of tumors isolated from tumor-bearing mice after control and MDSC-DCs ^Lym-del^ vaccines treated. *n* = 5 mice per group. * *p* < 0.05, ** *p* < 0.01, *** *p* < 0.001. Data were represented as mean ± standard deviation.

Next, we tried to figure out if depletion of CCL5 protein during the differentiation of MDSC-DCs could induce the formation of CD24 ^+^ MDSC-DCs, which would be important for CD24 ^+^ MDSC-DCs to be applied to clinical application. Previous studies have proved that CCL5 mainly expressed in T-lymphocyte subsets of blood cells (17,18); our reverse-transcription polymerase chain reaction (RT-PCR) results also showed that T-lymphocytes was the main source of CCL5 in splenocytes of tumor-bearing mice ( Fig. 6A ). We utilized murine CD3 microbeads to get rid of lymphocytes from splenocytes, then induced MDSC-DCs as previous described, and FACS results showed that lymphocyte-depleted MDSC-DCs displayed higher expression level of CD24 compared with control (CTR) MDSC-DCs ( Fig. 6B and 6 ).

To further confirm if the characteristics of lymphocyte-depleted MDSC-DCs were similar to those of CD24 ^+^ MDSC-DCs, we first examined the effect of these cells on Tregs polarization, and results showed that lymphocyte-depleted MDSC-DCs significantly inhibited the generation of Tregs, compared with control (CTR) MDSC-DCs, which was similar to that of CD24 ^+^ MDSC-DCs ( Fig. 6D and E ). Next, we tested the mRNA levels of Treg-polarization-related genes that had been demonstrated changed in CD24+ MDSC-DCs by quantitative PCR. The results showed that the expression of IL-12a, IL-12b, and CD24a was higher and the expression of CCL5, TGF-β1, and TGF-β3 was lower in lymphocyte-deplete MDSC-DCs compared with control cells ( Fig. 6F ), which were same tendency with that of CD24 ^+^ MDSC-DCs. At last, the therapeutic effect of lymphocyte-depleted MDSC-DCs based vaccine was tested in 4T1 tumor-bearing mice and these cells could significantly inhibit tumor growth, compared with control group ( Fig. 6G and H). These results suggested that CCL5-deficiency during in vitro differentiation could also induce similar cells as CD24+ MDSC-DCs.

### Decreasing the Level of CCL5 Protein Could Induce MDSCs from CRC Patients into CD24 ^+^ MDSC-DCs

**Fig. 7:**
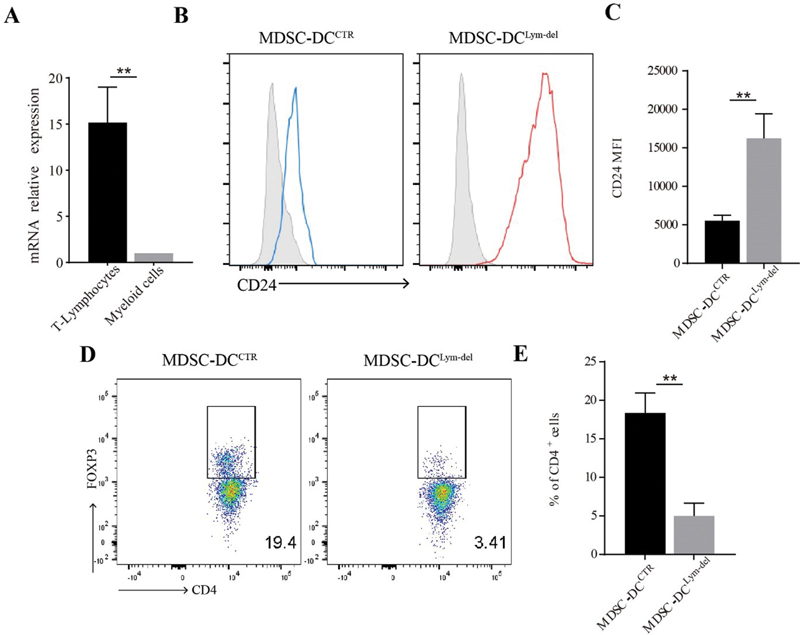
Myeloid-derived suppressive cells-derived DCs (MDSCs) from colorectal cancer (CRC) patients induced CD24 ^+^ MDSC-DC. ( **A** ) Relative gene expression of CC chemokine ligand 5 (CCL5) in T-lymphocytes and myeloid cells isolated from CRC cancer patients was examined by reverse-transcription polymerase chain reaction. ( **B** ) Representative expression of the indicated surface markers on gated CD11c ^+^ MDSC-DCs from CRC patients' peripheral blood mononuclear cell cultures. The isotype monoclonal antibody (mAb) of indicated markers was included as controls. ( **C** ) Mean Fluorescence Intensity (MFI) of CD24 in MDSC-DCs derived from CCL5 knockdown and control group. ( **D** ) MDSC-DCs cocultured with CD4 ^+^ T cells isolated from CRC patients peripheral blood for 7 days. The ratio of MDSCs and CD4 ^+^ T cells is 1 to 10 and CD4 ^+^ T cells were preactivated by CD3 and CD28 mAbs. After 7-day culture, the percentage of Tregs was analyzed by FACS. Representative staining plots of Tregs after 7-day coculture. ( **E** ) FACS qualification of the proportion of Tregs in the coculture system. The coculture experiment was repeated *n* = 3 for each group. * *p* < 0.05, ** *p* < 0.01, *** *p* < 0.001. Data were represented as mean ± standard deviation.

Next, we tried to figure out if depletion of CCL5 protein could induce tumor patients-derived MDSCs into CD24 ^+^ MDSC-DCs with similar antitumor function as murine CD24 ^+^ MDSC-DCs. For human CD24+ MDSC-DCs induction, we first collected peripheral blood samples of CRC patients in TNM Classification of Malignant Tumors (TNM) stage III or IV and enriched myeloid cells by Ficoll density gradient centrifugation. RT-PCR results also showed that CCL5 was mainly expressed in T-lymphocytes, but not myeloid cells **(** Fig. 7A ). To reduce the influence of CCL5 produced by lymphocytes, we depleted lymphocytes by human CD3 microbeads and utilized a similar method as what was used in induction of murine MDSC-DCs. FACS data showed that CD24 expression in MDSC-DC ^Lym-del^ was significantly higher than that of MDSC-DC ^CTR^ , indicating that CD24 ^+^ MDSC-DCS could be induced in vitro by getting rid of lymphocytes during the process of human MDSC-DCs differentiation ( Fig. 7B and C ).

To test whether CD24 ^+^ MDSC-DCS derived from CRC patients' peripheral blood could inhibit Tregs polarization, CD4 ^+^ T cells collected from the peripheral blood of tumor patients were preactivated by anti-CD3 and anti-CD28 and cocultured with human MDSC-DC _S_ for 7 days. FACS analysis showed that CD24 ^+^ MDSC-DCs from tumor patients could significantly inhibit CD4 ^+^ T cells from differentiating into Tregs ( Fig. 7D and E ). These data suggested that not only in mice model, but also in human samples, CD24 ^+^ MDSC-DCs with antitumor activity could be induced in vitro in absent of CCL5 protein, which provides a new method for DC-based vaccine development.

## Discussion

In this study, we revealed that CCL5-deficiency MDSCs could be induced into a new type of DCs with high CD24 expression (CD24 ^+^ MDSC-DCs), which had more antitumor activity than control MDSC-DCs. Antibody blocking and adoptive transfer experiments showed that Tregs mediated the antitumor activity of CD24 ^+^ MDSC-DCs. And CD24 ^+^ MDSC-DCs mainly inhibited Tregs' polarization, but not migration of Tregs. What's important, decreasing CCL5 protein in culture medium of both murine and human MDSCs during in vitro differentiation could also induce similar cells as CD24 ^+^ MDSC-DCs. These data suggest that knockdown of CCL5 levels in vitro might be used as a new method to induction of patients' peripheral blood monocytes or MDSCs derived DCs for tumor vaccines and gain higher antitumor activity.

Our previous study had shown that CCL5 could modulate the differentiation of MDSCs to promote tumor progression in luminal and triple-negative breast cancer. 22 38 And in CRC model, CCL5-deficiency inhibited tumor growth and metastasis by resulting in the metabolic disorders in CD11b ^hi^ F4/80 ^low^ TAMs and suppressing the expression of S100a9 to promote the migration of CD8 ^+^ T cells in the TME. 21 All of above studies suggested that CCL5 involved in differentiation of myeloid cells, besides its chemotaxis function. In this study, we further proved that CCL5 play an important role in the process of immature state of myeloid cells that differentiate into DCs. What's more, we found that ATRA, an inducer of myeloid differentiation, 26 27 could not only promote the differentiation of CCL5 ^−/−^ MDSCs and but also enhance the antitumor activity of CD24 ^+^ MDSC-DCS, suggesting that the signaling pathways between ATRA and CCL5 involved in induction of MDSC-DCs had some overlap.

CD24 is a glycosyl-phosphatidyl-inositol-anchored glycoprotein on the plasma membrane with a small protein core but extensive N-linked and O-linked glycosylation. CD24 is expressed by many immune cells 39 and previous studies showed that CD24 is primarily a costimulatory molecule for T lymphocyte activation in autoimmunity. 28 Recently, it was found that CD24 is often overexpressed in human tumors and CD24 on tumor cells was identified as an inhibitor of phagocytosis in “do not eat me” signal to play a suppressive role via binding to Siglec-10 on macrophages in tumor immunity. 40 However, what is the role of CD24 expression on MDSC-DCs? To prevent the phagocytosis of MDSC-DCs by other immune cells? Or to involve the polarization of CD4 T cells? The answers to this question might need our further studies to figure out in future study.

In conclusion, the antitumor activity of CD24 ^+^ MDSC-DCs was achieved by inhibiting the polarization of CD4 ^+^ T cells toward Tregs, therefore reducing the proportion of Treg in tumor and changing the immunosuppression of TME. In the process of inducing MDSC-DCs in vitro, we eliminated lymphocytes and massively obtained CD24 ^+^ MDSC-DCs with antitumor activity in the presence of ATRA. This improvement for DC vaccine provides a new idea for clinical transformation in the future to help tumor patients improve the efficacy of tumor treatment.

## Materials and Methods

### Mice and Cell Lines

BALB/c, C57BL/6, and OT-II transgenic mice designated B6.cgTg (TcraTcrb)425Cbn/J were purchased from the Jackson Laboratory, Bar Harbor, Maine, United States. CCL5-KO BALB/c and CCL5-KO C57/B6 were acquired as previously described. KO mice have no morphologically or functionally overt abnormal phenotype. Genotyping was done using PCR of tail DNAs. Mice were kept in pathogen-free conditions and handled in accordance with the requirements of the guideline for animal experiments.

The following s.c. tumor models were used: C57/B6 mice, B16F10 melanoma, MC38 colon carcinoma; BALB/c mice, 4T1 mammary carcinoma, CT26 colon carcinoma. All of these cell lines were purchased from American Type Culture Collection. The number of tumor cells injected was different for each model and was selected based on the ability to form tumor with 1.5 to 2 diameters within 3 to 4 weeks of injection.

### Generation of MDSC-DCs from Tumor Bearing Mice

Splenocytes were obtained from spleens of tumor-bearing mice. 10 × 106 splenocytes were cultured in Roswell Park Memorial Institute (RPMI) 1640 Medium supplemented with 10% fetal bovine serum, 20 ng/mL GM-CSF, 10 ng/mL IL-4, alone or in the presence of 1.5 μM ATRA. The culture was maintained at 37°C in 5% of CO _2_ -humified atmosphere in tissue-culture-treated 10 cm ^2^ dishes in 15 mL of medium. The culturemediumwas entirely disregarded at day 3 and replaced by fresh warm medium. On day 7, nonadherent cells in the culture supplement and loosely adherent cells harvested by gentle scraping with PBS were pooled and used as the starting source of material for most experiments.

### Tumor Digestion and Generation of Single-Cell Suspension

Tumor tissue (300–500 mg) was mechanically minced and digested in 10 mL PBS containing 0.5 mg/mL collagenase A, 0.01 mg/mL DNase I, and 10 U/mL hyaluronidase for 2 hours at 37°C and shaking at 80 rpm. The dissociated cells were collected, lysed by red blood cell lysis buffer. Then cell suspensions were filtered through a 70-μm nylon mesh. After centrifugation, cells were suspended in FACS buffer (2% (fetal calf serum [FCS], 2mM ethylenediaminetetraacetic acid [EDTA] in PBS) and immediately used for flow cytometry.

### Flow Cytometry

Cells that were obtained from blood, spleen, lymph, or tumor were stained in ice-cold PBS containing FCS (2%) and EDTA (2 mM) using appropriate antibody-fluorophore conjugates. The concentration of a single-cell suspension was adjusted to 1 × 10 ^6^ –10 ^7^ /mL. Multiparameter analysis was performed on a Fortessa analyzer (BD Biosciences) and analyzed with FlowJo software (Tree Star).

### In Vivo Depletion of Treg Cells

Treg depletion was performed by a single injection of 200ug purified anti-CD25 mAb(PC61). 4T1 bearing mice were treated intraperitoneally with anti-CD25 antibody at day 16 after tumor challenge.

### Adoptive Transfer of Isolated-Treg Cells

CD4+ CD25+ Tregs were purified from minced spleens obtained from female mice using magnetic bead separation (CD4+ CD25+ Treg kit, Miltenyi Biotec). Cells were cultured in RPMI-1640 media supplemented with 10% fetal bovine serum, 10,000 IU/mL penicillin, 10 mg/mL streptomycin, 1x GlutaMAX. Recombinant murine IL-2 were added at the concertation of 2,000 IU/mL. Tregs were activated with Dynabeads anti-CD3/CD28 CTS anti-CD3/anti-CD28-coated microbeads at a 2:1 bead/cell ratio. After 48-hour stimulation, magnetic beads were removed according to manufacturer's instruction and cells were harvested for adoptive transfer. At the onset of MDSC-DC treatment cases, 1 × 10 ^6^ iTregs were adoptively transferred into MDSC-DCs vaccine-recipient mice ( *n* = 5 per group) via the tail vein at day 16. Control mice were treated with PBS alone. All the mice were sacrificed at day 30 after tumor inoculation or the time when tumor volume reached 2 cm ^3^ .

### Murine naïve CD4+ T Cells Coculture with MDCS-DCs

Murine naïve CD4+ T cells were purified from minced spleens obtained from OT-II transgenic mice by Ficoll-hypaque solution and naïve CD4+ T cells isolation kit according to manufacturers' protocols. MDSC-DCs were cocultured with isolated CD4+ CD62L-OT-II cells in RPMI at 37°C, 5% CO _2_ in a ratio 1MDSC-DC: 10 T cells. When stated, 40 µg/mL OVA _323–339_ peptide, 100 ng/mL LPS, and 5ng/mL TGF-β1 were added. Cells were harvested after 4 days of coculture and analyzed by flow cytometry.

### Depletion of Lymphocytes by CD3 Microbeads

Splenocytes were harvested from 4T1 tumor-bearing mice and peripheral blood mononuclear cells were harvested from peripheral blood samples of CRC patients in TNM stage III or IV. T cells labeled with anti-CD3-coated beads were depleted with a commercially available magnetic separation kit (CD3 microbeads, Miltenyi Biotec) with either for human or for mouse.

### Quantitative RT-PCR

Samples for gene expression analysis were homogenized with 1mL TRI reagent (Invitrogen) to extract total RNA. cDNA was synthesized by reverse transcription of total RNA (Epicentre). Gene expression was probed using the following primer pairs: CCL5 (F, 5′-CAGTCGTCTTGTCACCCGA-3′; R, 5′-TGTAACTGCTGCTGTGTGGT -3′), TGFB1 (F, 5′-GAGTGGTTTGTTTGAGATGT-3′; R, 5′-GGTTCGTGCATCCATTTCCA -3′), TGFB3 (F, 5′-GGAAAACACCGAGTCGGAATAC-3′; R, 5′-GCGGAAAACCTTGGAGGTAAT-3′) IL12A (F, 5′-ACCACTCCCAAAACCTGC-3′; R, 5′-CCAGGCAACTCCCATTAG-3′) and IL12B (F, 5′-ACAAAGGAGGCGAGGTTCTG-3′; R, 5′-CTGTGGTCCATGCTGACTT-3′). GAPDH (F, 5′-GGAGCCAAAAGGGTCATCATCTC-3′; R, 5′-GAGGGGCCATCCACAGTCTTCT-3′) was used as a reference gene for normalization.

### Study Approval

All animal procedures were reviewed and approved by the Institutional Animal Care and Use Committee of Shanghai Jiao Tong University (BME (Ethics) 2017002).

All human samples were collected with the informed consent of the patients and the procedures were approved by Renji Hospital of Shanghai Jiao Tong University (Renji [2017] N017).

### Statistical Analysis

The Student's *t* -test was used to analyze the data. Results are given as mean ± standard error of mean unless otherwise indicated. *p* < 0.05 was considered statistically significant.
