# Supplementary material for: CD24 + MDSC-DCs Induced by CCL5-Deficiency Showed Improved Antitumor Activity as Tumor Vaccines
Source: Glob Med Genet. 2022 Mar 8;9(2):97–109. doi: 10.1055/s-0042-1743569 (PMC9192183; doi:10.1055/s-0042-1743569)
Supplement: Supplementary file 1 — Supplementary Material [file 10-1055-s-0042-1743569-s2200004.pdf]

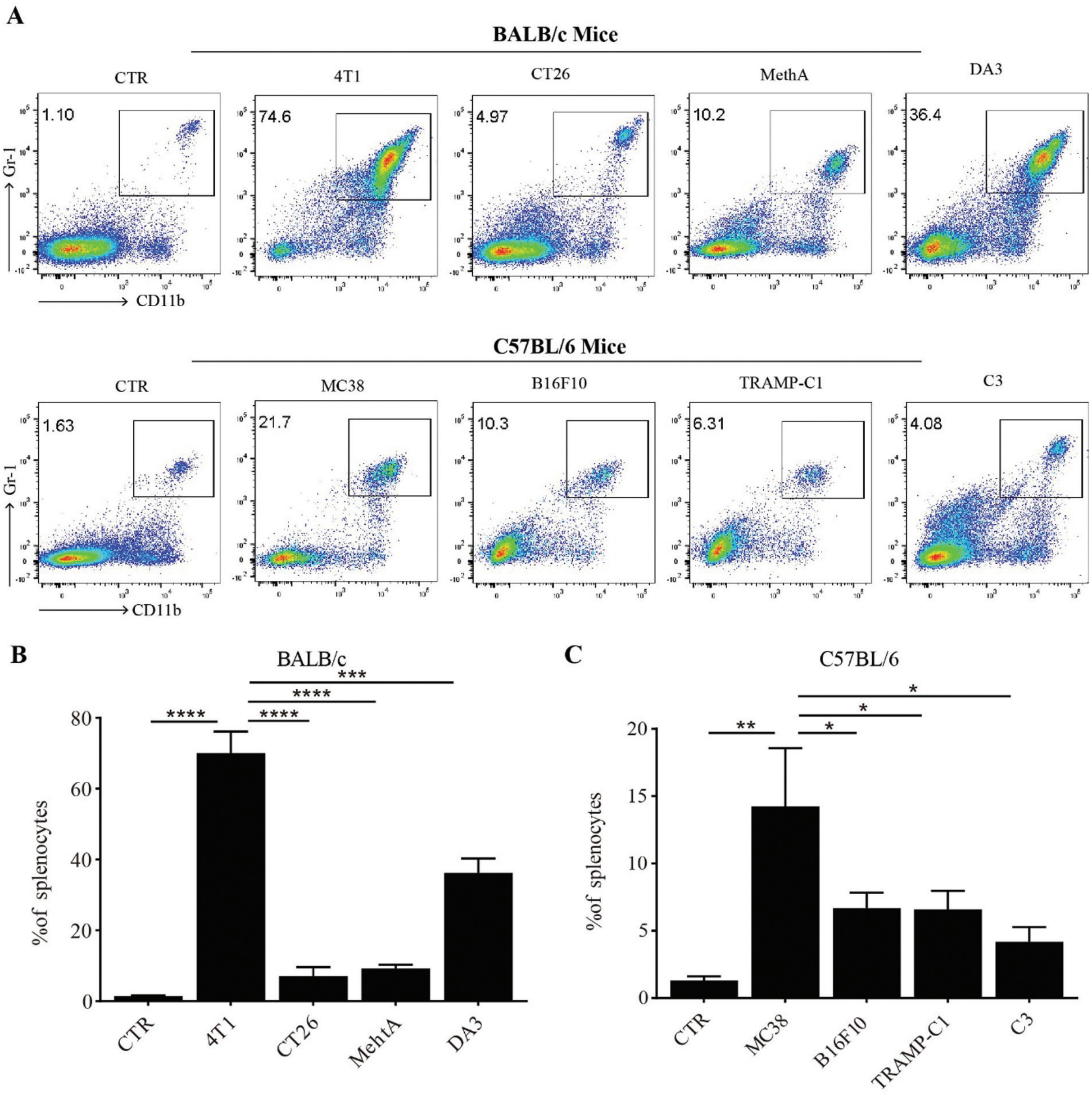

Supplementary Fig. S1 Analyzed percentage of Gr-1^{+} CD11b^{+} myeloid-derived suppressive cells-derived DCs in spleens of tumor bearing mice by FACS. (**A**) Typical example of flow cytometry analysis. (**B**) The percentage of Gr-1^{+} CD11b^{+} cells in spleen on the BALB/c backgrounds as indicated. Each group included from $n = 5$ mice. Differences between the proportion of Gr-1+ CD11b+ cells in spleens from naive and tumor-bearing mice were statistically significant for all tumor models. (**C**) The percentage of Gr-1^{+} CD11b^{+} cells in spleen on the C57BL/6 backgrounds as indicated. Each group included from $n = 5$ mice. Differences between the proportion of Gr-1+ CD11b+ cells in spleens from naive and tumor-bearing mice were statistically significant for all tumor models. $^{*}p < 0.05$, $^{**}p < 0.01$, $^{***}p < 0.001$, $^{****}p < 0.0001$. Data were represented as mean ± standard deviation.

 © 2022. The Author(s).

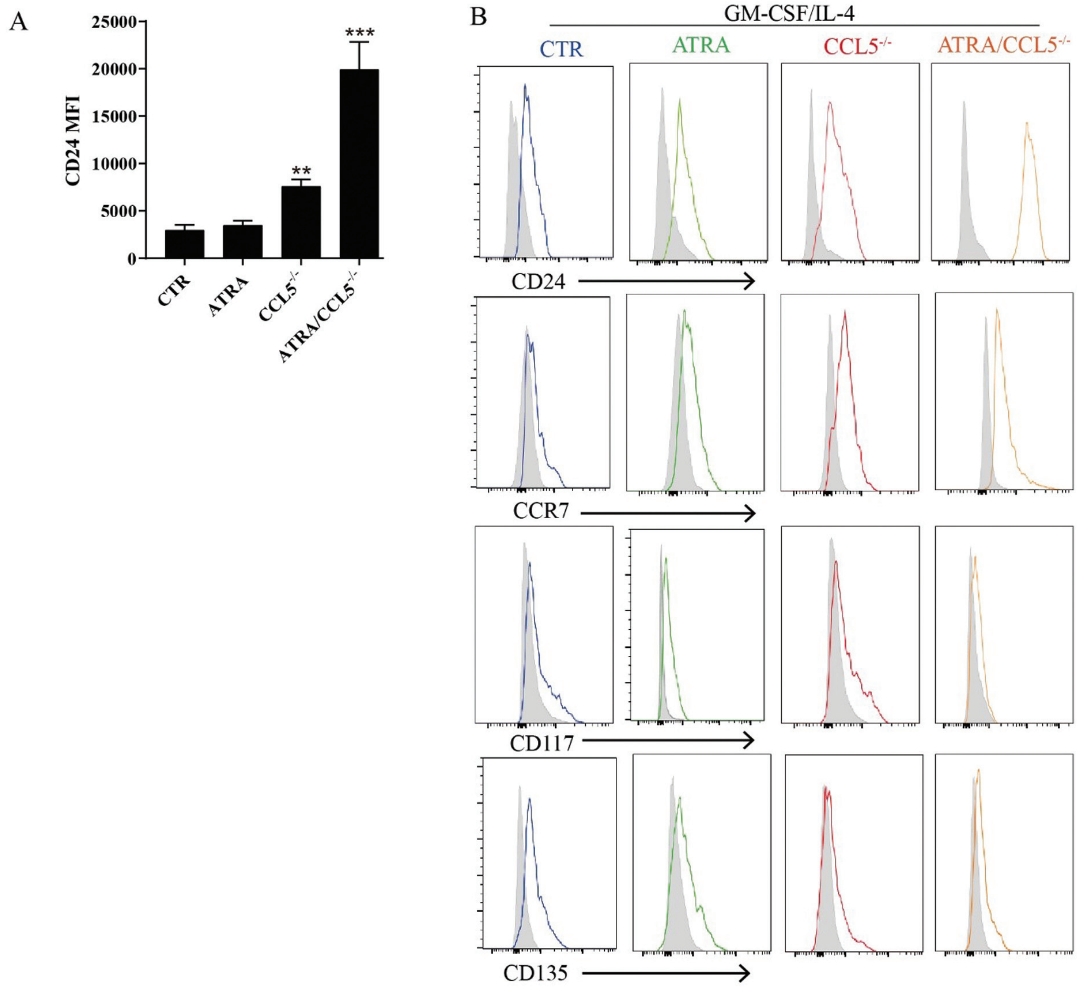

Supplementary Fig. S2 Analyzed dendritic cell markers expression of myeloid-derived suppressive cells-derived DCs (MDSC-DCs) in MC38 tumor bearing mice by FACS. (**A**) Representative expression of the indicated surface markers on gated CD11c^{+} MDSC-DCs from MC38 tumor-bearing mice splenocytes cultures. The isotype monoclonal antibody of indicated markers were included as controls. (**B**) CD24 expression in MDSC-DCs derived from different culture methods. The experiments were repeated $n = 3$ for each group. All the significance in the plot were compared with control (CTR) group. $^{*}p < 0.05$, $^{**}p < 0.01$, $^{***}p < 0.001$. Data were represented as mean ± standard deviation. ATRA, all-trans-retinoic acid; CCL5$^{-/-}$, CC chemokine ligand 5$^{-/-}$; GM-CSF/IL-4, granulocyte macrophage–colony stimulating factor/interleukin-4.

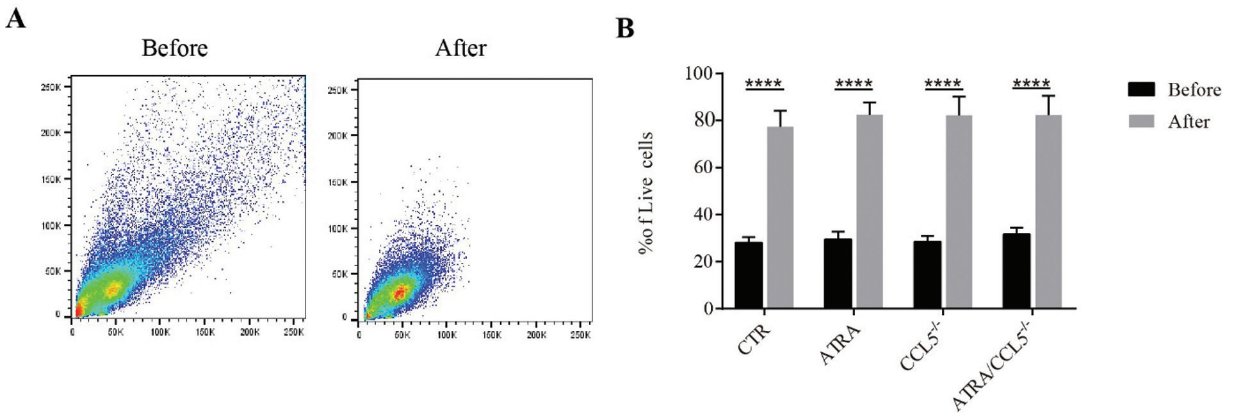

Supplementary Fig. S3 Cell debris and unwanted cells were removed in myeloid-derived suppressive cells-derived DC (MDSC-DC) cultures by debris removal solution and Ficoll. (**A**) Representative flow cytometry analysis of purified MDSC-DCs in different culture groups. (**B**) FACS qualification of CD45^{+} cells in different groups before/after process. The experiments were repeated $n = 3$ for each group. $^{*}p < 0.05$, $^{**}p < 0.01$, $^{***}p < 0.001$,$^{****}p < 0.0001$. Data were represented as mean ± standard deviation. ATRA, all-trans-retinoic acid; CCL5$^{-/-}$, CC chemokine ligand 5$^{-/-}$.

 © 2022. The Author(s).

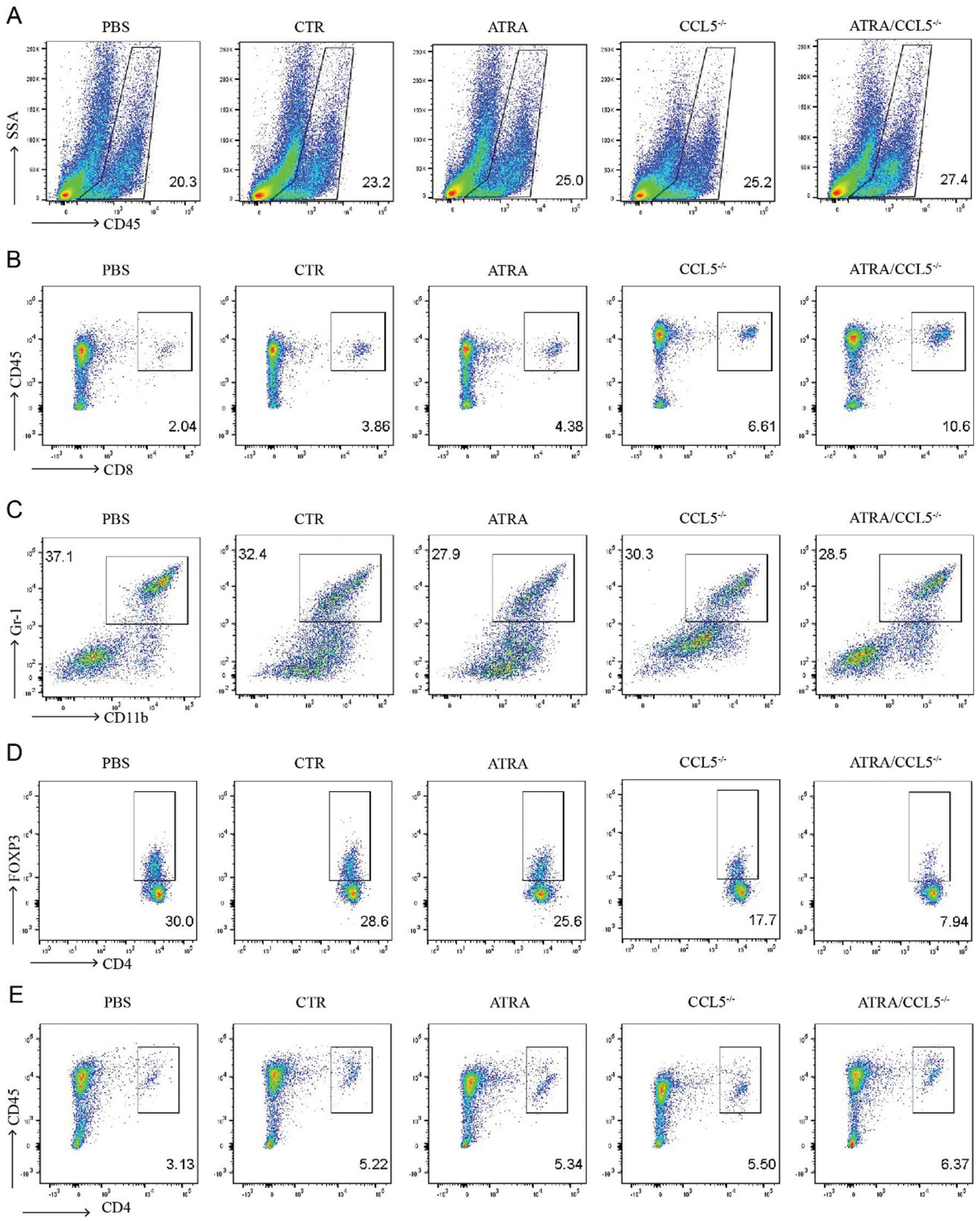

Supplementary Fig. S4 The number of tumor infiltrated immune cells were changed after myeloid-derived suppressive cells-derived DCs (MDSC-DCs) vaccines treatment. (**A**) Representative staining plots of tumor infiltrated CD45$^+$ cells in different MDSC-DC vaccines treatment groups. (**B**) Representative staining plots of tumor infiltrated CD8$^+$ cells in different MDSC-DCs vaccines treatment groups. (**C**) Representative staining plots of tumor infiltrated MDSCs in different MDSC-DCs vaccines treatment groups. (**D**) Representative staining plots of tumor infiltrated CD4$^+$ cells in different MDSC-DCs vaccines treatment groups. (**E**) Representative staining plots of tumor infiltrated regulatory T cells in different MDSC-DCs vaccines treatment groups. ATRA, all-trans-retinoic acid; CCL5$^{-/-}$, CC chemokine ligand 5$^{-/-}$; PBS, phosphate buffered saline.

 © 2022. The Author(s).
